# Ruxolitinib inhibits cytokine production by human lung macrophages without impairing phagocytic ability

**DOI:** 10.3389/fphar.2022.896167

**Published:** 2022-08-19

**Authors:** Nikola Mantov, Mathilde Zrounba, Marion Brollo, S Grassin-Delyle, Matthieu Glorion, Mélanie David, Emmanuel Naline, Philippe Devillier, Hélène Salvator

**Affiliations:** ^1^ Laboratory of Research in Respiratory Pharmacology—Virologie et Immunologie Moleculaire (VIM) Suresnes, V2I—UMR-0892 Paris Saclay University, Suresnes, France; ^2^ Respiratory Diseases Department, Foch Hospital, Suresnes, France; ^3^ Infection and Inflammation, Health Biotechnology Department, Paris-Saclay University, UVSQ, INSERM, Montigny le Bretonneux, France; ^4^ Thoracic Surgery Department, Foch Hospital, Suresnes, France; ^5^ Faculté des Sciences de la Santé Simone Veil, UVSQ Paris-Saclay University, Montigny-le-Bretonneux, France

**Keywords:** ruxolitinib, human macrophages, Janus kinase (2)-signal transducers and activators of transcription [JAK2-STAT5], cytokine, budenoside

## Abstract

**Background:** The Janus kinase (JAK) 1/2 inhibitor ruxolitinib has been approved in an indication of myelofibrosis and is a candidate for the treatment of a number of inflammatory or autoimmune diseases. We assessed the effects of ruxolitinib on lipopolysaccharide (LPS)- and poly (I:C)-induced cytokine production by human lung macrophages (LMs) and on the LMs’ phagocytic activity.

**Methods:** Human LMs were isolated from patients operated on for lung carcinoma. The LMs were cultured with ruxolitinib (0.5 × 10^−7^ M to 10^–5^ M) or budesonide (10^–11^ to 10^–8^ M) and then stimulated with LPS (10 ng·ml^−1^) or poly (I:C) (10 μg·ml^−1^) for 24 h. Cytokines released by the LMs into the supernatants were measured using ELISAs. The phagocytosis of labelled bioparticles was assessed using flow cytometry.

**Results:** Ruxolitinib inhibited both the LPS- and poly (I:C)-stimulated production of tumor necrosis factor alpha, interleukin (IL)-6, IL-10, chemokines CCL2, and CXCL10 in a concentration-dependent manner. Ruxolitinib also inhibited the poly (I:C)- induced (but not the LPS-induced) production of IL-1ß. Budesonide inhibited cytokine production more strongly than ruxolitinib but failed to mitigate the production of CXCL10. The LMs’ phagocytic activity was not impaired by the highest tested concentration (10^–5^ M) of ruxolitinib.

**Conclusion**: Clinically relevant concentrations of ruxolitinib inhibited the LPS- and poly (I:C)-stimulated production of cytokines by human LMs but did not impair their phagocytic activity. Overall, ruxolitinib’s anti-inflammatory activities are less intense than (but somewhat different from) those of budesonide—particularly with regard to the production of the corticosteroid-resistant chemokine CXCL-10. Our results indicate that treatment with a JAK inhibitor might be a valuable anti-inflammatory strategy in chronic obstructive pulmonary disease, Th1-high asthma, and both viral and non-viral acute respiratory distress syndromes (including coronavirus disease 2019).

## Introduction

Many cytokines and growth factors rely on the Janus kinase-signaling transducer and activator of transcription proteins (JAK-STAT) pathway for signal transmission (Heim 1999; Hu et al., 2021). Four members of the JAK family (JAK1, JAK2, JAK3, and TYK2) elicit the phosphorylation of seven STAT proteins (STAT1, 2, 3, 4, 5A, 5B, and 6) ubiquitously expressed in mammalian cells. JAK/STAT signaling is involved in various cellular processes, including innate immune responses, tumor cell growth, and autoimmunity (Hu et al., 2021).

Accordingly, the identification and development of JAK inhibitors for the treatment of tumoral, inflammatory and autoimmune diseases has attracted much interest in recent years (Tzeng and Tsu Chyuan, 2021; Clark and Flanagan, 2014). Ruxolitinib is a JAK inhibitor that targets JAK1 and two preferentially. The drug is currently indicated for the treatment of intermediate- or high-risk primary myelofibrosis and post-polycythemia vera myelofibrosis (Verstovsek et al., 2012; Vannucchi et al., 2015). Ruxolitinib also has proven efficacy in the management of severe, corticosteroid-resistant graft-versus-host disease following allogeneic bone marrow transplantation (Zeiser et al., 2020, Zeiser et al., 2021). More generally, JAK targeting is a therapeutic strategy of interest in many chronic inflammatory diseases, such as rheumatoid arthritis (with the JAK3 inhibitor tofacitinib) (Strand et al., 2015).

With regard to the various cell types in the lung, the interferon (IFN)-γ-induced JAK/STAT signaling pathways are corticoid-insensitive in alveolar macrophages and epithelial cells (Southworth et al., 2012; O’Connell et al., 2015). Furthermore, the inhibition of JAK1 signaling reduced IFN-γ-stimulated CXCL10 responses and IFN-γ-enhanced lipopolysaccharide (LPS) responses in alveolar macrophages (Southworth et al., 2012). Accordingly, JAK inhibitors (including ruxolitinib) might constitute a new option for the treatment of inflammatory lung diseases (Wang et al., 2020) like COPD (Southworth et al., 2012; Yew-Booth et al., 2015), severe asthma (O’Connell et al., 2015; Subramanian et al., 2021), severe sarcoidosis (Rotenberg et al., 2018), scleroderma (Lescoat et al., 2020), and rare interferonopathies (Frémond et al., 2020).

Given the JAK-STAT pathway’s role for many cytokines and growth factors involved in the immune response and lung homeostasis, JAK-STAT inhibition can also lead to pulmonary adverse events. Safety data from clinical trials and meta-analyses have highlighted the elevated risk of infectious complications in patients on ruxolitinib (Khalid et al., 2021; Tsukamoto et al., 2018; Wysham and Sullivan, 2013; Sayabovorn and Chongtrakool, 2021). Furthermore, we have reported on suspected cases of ruxolitinib-induced pulmonary alveolar proteinosis in recipients of hematopoietic stem cell allografts (Salvator et al., 2018; Salvator Hélène et al., 2021).

The macrophages are the most abundant immune cells in the lung and are involved in the pathophysiology of acute and chronic pulmonary inflammatory diseases. To the best of our knowledge, ruxolitinib’s direct effects on human LMs have not previously been explored. The objective of the present study was to investigate ruxolitinib’s impact on LM activation in terms of cytokine production in response to LPS and poly (I:C) and phagocytic activity. LPS, agonist of Toll Like Receptor (TLR) 4, is usually used as classically-activated polarization agent for macrophage and Poly (I:C), TLR3 agonist, has been chosen for its ability to mimic an activation of the cells by a viral attack (Grassin-Delyle et al., 2020). Corticosteroids are the corner stone of the treatment of many chronic bronchial diseases (COPD, asthma) and used in pulmonary acute inflammation induced by viral infection (COVID-19). Because of the specificity of the JAK/STAT pathways towards corticosteroids effect (Southworth et al., 2012; O’Connell et al., 2015), we chose to compare the expected effects of ruxolitinib to the one of budenoside.

## Material and methods

### Materials

Ruxolitinib was obtained from Selleck Chemicals LLC (Houston, TX), and budesonide was obtained from Sigma (St. Louis, MO). Both were dissolved in vehicle (0.05% dimethylsulfoxide), which did not interfere with cytokine production (data not shown). Antibiotics, dimethylsulfoxide, L-glutamine, trypan blue dye, heat-inactivated fetal calf serum, and LPS (from *E. coli* serotype 0111:B4) were purchased from Sigma. High-molecular-weight poly (I:C) was obtained from InvivoGen (Toulouse, France). Bovine serum albumin and Roswell Park Memorial Institute (RPMI) medium were purchased from Eurobio Biotechnology (Les Ulis, France).

### Preparation of human LMs

Experiments on human tissue were approved by the regional investigational review board (*Comité de Protection des Personnes Île de France VIII*, Boulogne-Billancourt, France). Lung tissue samples were obtained from 23 patients [mean ± standard error of the mean (SEM) age: 64.4 ± 11.4; 11 males and 12 females; Forced Expiratory Volume in 1 s (FEV1)/Forced Vital Capacity (FVC) (%): 67.5 ± 12.0; (smokers: 18/23, including COPD: 11) undergoing surgical resection for lung carcinoma and who had not received prior chemotherapy. In line with the French legislation on clinical research and as approved by the investigational review board, all the patients gave their informed consent for the use of resected lung tissue for *in vitro* experiments.

Lung macrophages were isolated by adherence, as described previously (Grassin-Delyle et al., 2019; Salvator Helene et al., 2021). Briefly, the fluid collected from several washings of minced peripheral lung tissues was centrifuged (2,000 rpm for 10 min). The cell pellet was resuspended in RPMI medium supplemented with 10% heat-inactivated fetal calf serum, 2 mM L-glutamine, and antibiotics. Viable cells (10^6^ per mL) were seeded into a 24-well plate (for ELISA assays) or a 12-well plate (for phagocytosis experiments). Following incubation for at least 1.5 h at 37°C (in a humidified 5% CO_2_ atmosphere), non-adherent cells were removed by gentle washing. The remaining cells were maintained at 37°C with 5% CO_2_ overnight. As described in previous reports from our group (Buenestado et al., 2010, Buenestado et al., 2012; Abrial et al., 2015; Victoni et al., 2017), 95% or more of the adherent cells (mean ± SEM cells per well in a 24-well plate: 215 ± 18 × 10^3^) were macrophages, as determined by May-Grünwald-Giemsa staining and CD68 immunocytochemistry (data not shown). Cell viability exceeded 90%, as assessed in a trypan blue dye exclusion assay. Culture plates with adherent macrophages were washed with warm medium. One mL of fresh medium supplemented with 1% heat-inactivated fetal calf serum was added per well, and culture plates were incubated overnight at 37°C in a 5% CO_2_ humidified atmosphere.

### Treatment of LMs

On the day after isolation, macrophages were washed twice, and 1 ml of RPMI medium with 1% fetal calf serum was added per well. The LMs were exposed for 24 h to LPS (10 ng·ml^−1^) or heat-inactivated poly (I:C) (10 μg·ml^−1^). On the basis of time-response and concentration-response curves from preliminary experiments, we deliberately selected a suboptimal LPS concentration (10 ng·ml^−1^) (Buenestado et al., 2012). The poly (I:C) concentration (10 μg·ml^−1^) was also selected on the basis of previous experiments (Salvator Hélène et al., 2021). Ruxolitinib (5 × 10^−7^, 10^–6^, 5 × 10^−6^, 10^–5^ M) or budesonide (10^–11^, 10^–10^, 10^–9^, and 10^–8^ M) was added to the culture medium 1 h before exposure to LPS or poly (I:C). The ruxolitinib concentrations used *in vitro* were initially chosen by reference to those measured in the plasma of treated patients; these have been shown to be effective *in vitro* on monocytes (Lescoat et al., 2020; Isberner et al., 2021). After a 24-hour incubation, the remaining cells were counted in each plate and the supernatants were collected and stored at −20°C for subsequent analysis.

### ELISAs and cytotoxic effect

The cytokine concentrations in the supernatants were measured with ELISAs (R&D Systems, Minneapolis, MN), according to the manufacturer’s instructions. The respective assays’ limits of detection were 1.9 pg·ml^−1^ for interleukin (IL)-1β, 31.2 pg·ml^−1^ for IL-10, 7.8 pg.ml^−1^ for tumor necrosis factor alpha (TNF-α) and CCL2, 9.4 pg·ml^−1^ for IL-6, and 15.6 pg·ml^−1^ for CXCL10. The supernatants were diluted with reagent diluent as required, and the optical density was determined at 450 nm using a microplate reader (MRX II, Dynex Technologies, Saint-Cloud, France). Amounts of cytokine were reported to the numbers of remaining attached cells and expressed in pg.10^−6^ LMs.

Cytotoxicity was determined by measuring the lactate dehydrogenase activity in the LM supernatants, using the CytoTox96^®^ Non-Radioactive Cytotoxicity Assay (Promega, Madison, WI) after 24 h of exposure to ruxolitinib at the highest concentration (10^−5^ M) Additionally, the percentage of live cells was determined by staining the LMs cultured with ruxolitinib (10^−5^ M) during 24 h with a Live/dead stain (LIVE/DEAD Fixable Aqua Dead Cell Stain Kit, ThermoFisher Scientific, Waltham, MA).

### Flow cytometry evaluation of phagocytosis

The LMs’ phagocytic activity was assessed using *E. coli* bioparticles coupled to fluorochrome Alexa Fluor 488 (ThermoFisher Scientific, Waltham, MA). The day after isolation, the LMs were treated with ruxolitinib (10^–5^ M). After 24 h of incubation, the culture medium was replaced with 500 ml of FACS Buffer. After centrifugation and sonication, the bioparticles were opsonized and added to the cell culture at a concentration of 10 bioparticles per LM. The LMs were then incubated in the dark for 1 h at 37°C or (as a negative control) 4°C and then washed twice with PBS at 4°C to stop the phagocytosis. The LMs were detached by successive rounds of washing and resuspension in cytometry tubes in FACS buffer before being fixed in a 1% paraformaldehyde solution. Phagocytic activity was measured using fluorescence flow cytometry (LSR Fortessa, Becton-Dickinson, Franklin Lakes, NJ) and analyzed with FlowJo software (version 10.4, Becton-Dickinson).

### Statistical analysis

The effects of ruxolitinib (10^−5^ M) on basal cytokine production and the effects of LPS or poly (I:C) on cytokine production by LMs were assessed using paired or unpaired Student’s *t* tests, as appropriate. The effects of ruxolitinib or budesonide on LPS- or poly (I:C)-stimulated cytokine production by paired LM preparations were analyzed in either an analysis of variance (ANOVA) with repeated measures and Bonferroni’s post-test (for normally distributed data) or Friedman’s test with Dunn’s post-test (for non-normally distributed data). Data were expressed as the mean ± SEM amount of cytokine production or as a percentage of the LPS- or poly (I:C)-stimulated production of cytokine per 10^6^ LMs obtained from n patients. The threshold for statistical significance was set to *p* < 0.05. All statistical analysis were performed using GraphPad Prism^®^ software (version 7, GraphPad Software Inc., San Diego, CA).

## Results

### Ruxolitinib abrogates the LPS- and poly (I:C)-induced production of cytokines by LMs

The incubation with ruxolitinib (10^−5^ M) in presence or not of LPS or Poly (I:C) was not associated with a significant increase in LDH release by the LMs (*n* = 5) (CytoTox96^®^ Non-Radioactive Cytotoxicity Assay, Promega^®^). The absence of significant of Ruxolitinib on cellular viability has also been checked by Live/dead staining and flow cytometry analysis (Supplementary Table S1; Supplementary Figure S1).

#### Effect of ruxolitinib on cytokine production by non-stimulated LMs

Ruxolitinib did not have a significant impact on the basal release of cytokines by non-stimulated LMs (Table 1).

**TABLE 1:** Amounts of cytokines in the supernatants of non-stimulated human LMs treated for 24 h with vehicle (basal release) or 10^–5^ M ruxolitinib.

	Basal release	Ruxolitinib 10^–5^ M
IL-1β *n* = 7	19.9 ± 15.7	27.9 ± 24.4
TNF-α *n* = 10	415.1 ± 190.5	454.9 ± 285.4
IL-6 *n* = 8	344.1 ± 185.2	574.9 ± 292.1
CCL2 *n* = 10	2,871.2 ± 722.2	2,435.2 ± 621.4
CXCL10 *n* = 11	143.7 ± 76.5	88.1 ± 46.2
IL-10 *n* = 11	220.1 ± 180.1	280.4 ± 176.2

The mean ± SEM results of 7–11 independent paired experiments are expressed in pg.10^−6^ LMs.

#### LPS- and poly (I:C)-induced cytokine production by LMs

Incubation with LPS or poly (I:C) for 24 h was associated with markedly greater production of the assayed cytokines, relative to basal conditions. Exposure to LPS was associated with greater production of TNF-α, IL-6, and IL-10, while exposure to poly (I:C) was associated with greater production of CXCL10 and IL-1β (Table 2).

**TABLE 2:** Amounts of cytokines in the supernatants of human LMs treated for 24 h with LPS or poly (I:C).

	Basal release	poly (I:C) 10 μg·ml^−1^	LPS 10 ng·ml^−1^	LPS vs. poly (I:C) fold change
IL-1β *n* = 8–15	17.4 ± 6.8	872.5 ± 291.7 [50.1]*	342.6 ± 79.2 [19.7]***	0.4*
TNF-α *n* = 10–16	313.2 ± 117.1	7,793.9 ± 2,268 [24.9]**	50,456.9 ± 6,025.5 [161.1]***	6.5***
IL-6 *n* = 8–16	253.8 ± 105.6	5,615.9 ± 2,443.8 [21.0]**	79,618.1 ± 15658.4 [297.1]***	13.1**
CCL2 *n* = 6–16	4,304.7 ± 802.1	13858.1 ± 3,472.5 [3.2]*	34,037.4 ± 6,132.1 [7.9]***	2.5 ns
CXCL10 *n* = 14–16	75.2 ± 22.2	6,849.2 ± 1,321.3 [91.0]***	3,186 ± 788.8 [42.3]**	0.5*
IL-10 *n* = 7–9	228.9 ± 58.9	642.2 ± 128.7 [2.8]*	2,375.4 ± 296.7 [10.4]***	3.7***

The mean ± SEM results of six to 16 independent experiments are expressed in pg.10^−6^ LMs. The increase in cytokine production induced by LPS or poly (I:C) is also expressed as the mean fold-change vs. basal conditions and is given in square brackets. Asterisks indicate a significant difference vs. basal conditions or a significant difference between LPS and poly (I:C) (*: <0.05; **: <0.01; ***: <0.001).

#### Effect of ruxolitinib on the LPS- and poly (I:C)-induced production of cytokines by LMs

**FIGURE 1:**
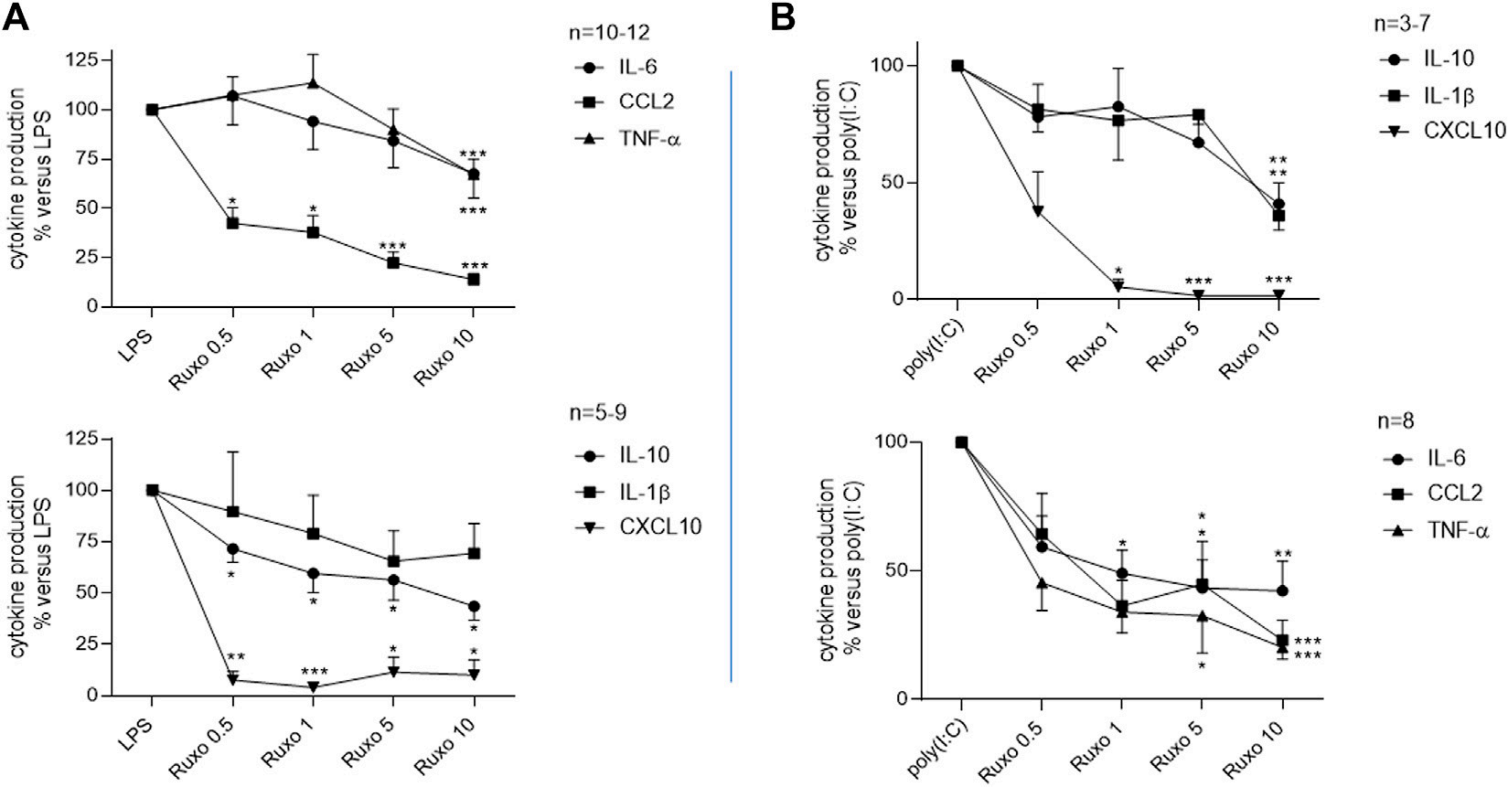
Ruxolitinib’s inhibitory effect on the LPS or poly (I:C)-induced production of cytokines by LMs. LMs were treated with ruxolitinib (0.5 × 10^–6^ to 10 × 10^–6^ M) before stimulation with **(A)**: LPS (10 ng·ml^−1^) or **(B)**: poly (I:C) (10 μg·ml^−1^) for 24 h. Data are expressed as a percentage of LPS or poly (I:C)-induced levels of cytokine production. The mean ± SEM results of eight to 12 independent experiments are shown. Asterisks indicate significant differences with respect to LPS or poly (I:C) (*: <0.05; **: <0.01; ***: <0.001).

Ruxolitinib inhibited the LPS-induced production of TNF-α, IL-6, CCL2, CXCL10, and IL-10 but failed to significantly reduce the production of IL-1ß (Figure 1; Supplementary Table S2). The drug also inhibited the poly (I:C)-induced production of IL-1ß, TNF-α, IL-6, CCL2, CXCL10, and IL-10 in a concentration-dependent manner (Figure 1; Supplementary Table S3). Ruxolitinib’s inhibitory effect was greatest on CCL2 and CXCL10, the production of which fell by 80%–98%, and expressed in a concentration dependent manner on the amount of these two cytokines.

#### Effect of budesonide on LPS-induced cytokine production by LMs

**FIGURE 2:**
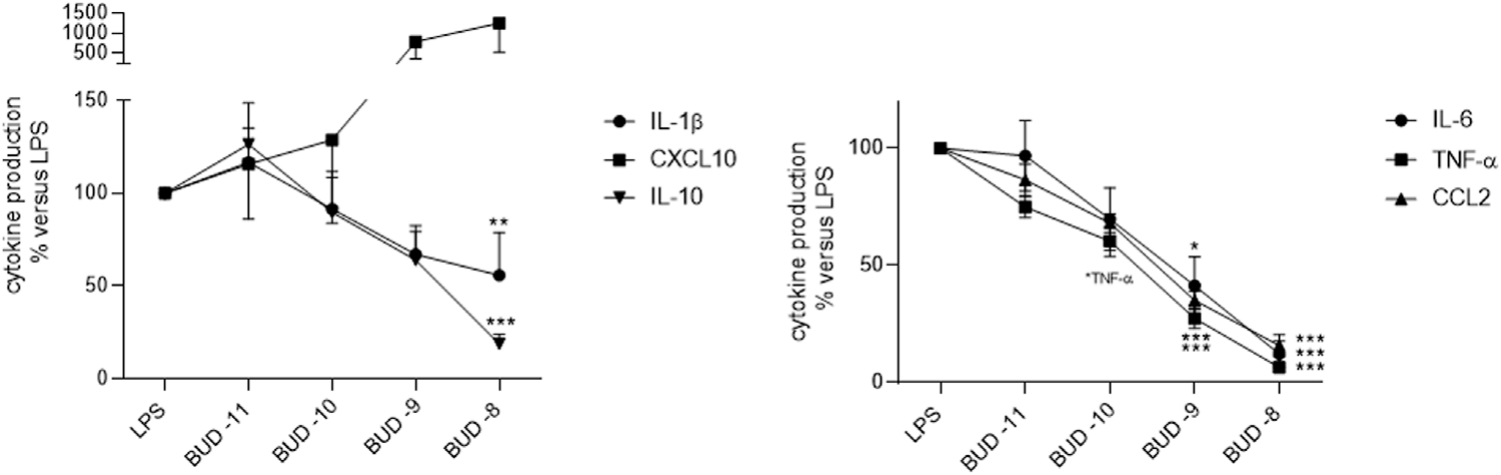
Effect of budesonide on the LPS-induced production of cytokines by LMs. The cells were treated with budesonide (at concentrations ranging from 10^–11^ to 10^–8^ M) before stimulation with LPS (10 ng·ml^−1^) for 24 h. The mean ± SEM results of five to eight independent experiments are expressed as a percentage of the response to LPS. Asterisks indicate significant differences relative to LPS (*: <0.05; **: <0.01; ***: <0.001).

Budesonide inhibited the LPS-induced production of IL-1ß, TNF-α, IL-6, CCL-2, and IL-10 in a concentration-dependent manner (Figure 2; Supplementary Table S4). At the highest concentration tested (10^–8^ M), budesonide abrogated the LPS-stimulated production of these five cytokines. However, budesonide treatment of LMs stimulated the production of CXCL10.

### Effect of ruxolitinib on the LMs’ phagocytic activity

**FIGURE 3:**
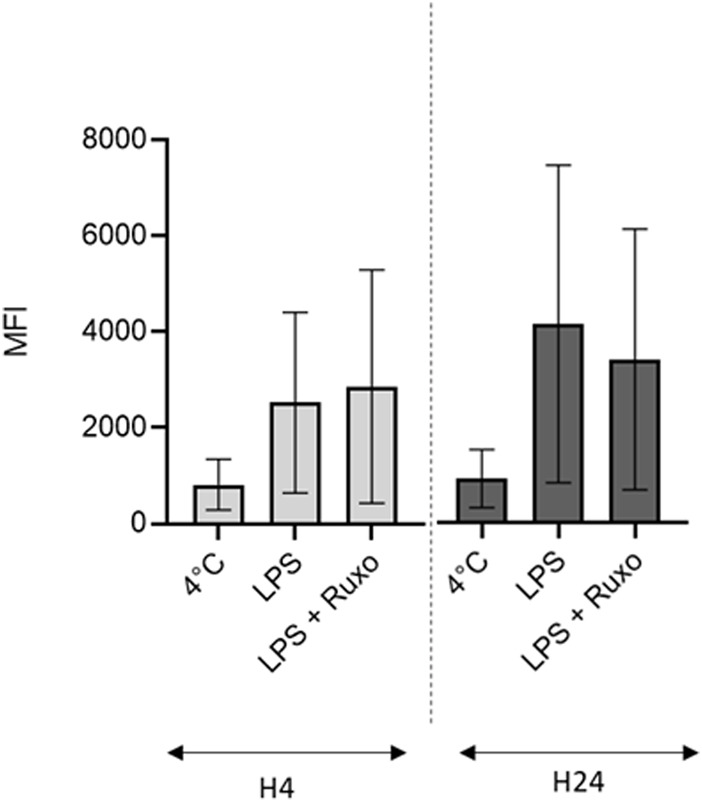
Ruxolitinib’s effect on the phagocytic activity of LMs. The day after isolation, LMs were treated with ruxolitinib (10^–5^ M) in the presence of LPS (10 ng/ml) for 4 and 24 h, before being exposed to fluorescent, opsonized *E. coli* bioparticles (10 bioparticles per LM). The intensity of intracellular fluorescence after exposure to ruxolitinib was compared with those for LMs stimulated with LPS alone (positive control) and for LMs incubated at 4°C (negative control). The results of three independent experiments are represented graphically as median (bar) and range. MFI: mean fluorescence intensity. Number of events was on average 6,653 ± 326.

In a flow cytometry assessment of ruxolitinib’s impact on the LMs’ phagocytic activity, there was no difference vs. the positive control—even at the highest concentration (10^–5^ M) in inflammatory conditions, after short (4 h) or long term (24 h) exposure to LPS. Hence, ruxolitinib did not alter the macrophages’ phagocytic activity (Figure 3).

## Discussion

### Main findings

The present study is the first to have addressed the impact of the JAK1/2 inhibitor ruxolitinib on human LMs. Our results demonstrated that ruxolitinib exerted an anti-inflammatory effect by reducing the LPS-and poly (I:C)-stimulated production of cytokines. Importantly, however, ruxolitinib did not impair the LMs’ phagocytic activity *in vitro*.

### Ruxolitinib’s anti-inflammatory effects on preparations of murine and human macrophages

Our observation of ruxolitinib’s anti-inflammatory effects on human LMs extends the literature data on the effects of JAK inhibitors on murine macrophage cell lines and bone-marrow-derived macrophages (BMDMs), and on human blood monocyte-derived macrophages (MDMs).

AG490 (a specific JAK2 inhibitor) inhibited the LPS-induced production of IL-1β, IL-6, and TNF-α by the RAW264.7 murine macrophage-like cell line (Okugawa et al., 2003; NamOh et al., 2012) and the LPS-induced gene expression of IL-6, IL-12p40, IL-1β, and CXCL10 in mouse primary BMDMs (FreitasMaria Cecilia et al., 2010). Ruxolitinib also inhibited the LPS-induced mRNA expression and protein production of CCL2 in primary BMDMs from IL-10 knock-out mice (Pattison and MacKenzieElcombe, 2013).

In human MDMs, ruxolitinib downregulated the mRNA expression and protein production of most of the LPS-induced cytokines—including those assessed in the present study (TNF-α, IL-6, CCL2, and CXCL10). Although ruxolitinib fully repressed the LPS-induced mRNA expression of most cytokines (including CCL2 and CXCL10), the repression was partial for IL-6 and TNF-α and null for IL-1β and CXCL2 (Febvre-James et al., 2018). Accordingly, in studies of MDM culture supernatants, ruxolitinib exerted a greater inhibitory effect on the LPS-induced production of CCL2 and CXCL10 than on the production of TNF-α and IL-6 (Febvre-James et al., 2018). Furthermore, three JAK inhibitors (including ruxolitinib) inhibited the production of TNF-α, CXCL10, and IL-6 in human MDMs activated by IFNγ (alone or in combination with LPS) (Lescoat et al., 2020). However, only ruxolitinib significantly inhibited the production of these three cytokines in MDMs activated by IFNγ and LPS (Lescoat et al., 2020). Lastly, ruxolitinib inhibited the expression of CXC10 and CXCL11 by MDMs from healthy subjects and by macrophages isolated from the synovial fluid of patients with rheumatoid arthritis (Yarilina et al., 2012).

### Key features of ruxolitinib’s anti-inflammatory effects on human LMs

In the present study of human LMs, ruxolitinib’s inhibitory effect on LPS-induced cytokine production was greater for CCL2 and CXCL10 than for IL-1ß, TNF- α, IL-6, and IL-10, as previously reported for MDMs (particularly at the mRNA level) (Febvre-James et al., 2018). In the poly (I:C)-stimulated LMs, ruxolitinib’s inhibitory effect on cytokine production was greater for CXCL10 than for TNF-α, IL-6, and CCL2, and the production of IL-1ß and IL-10 was inhibited only at the highest tested concentration of ruxolitinib (10^–5^ M). The results with poly (I:C) extend our knowledge of ruxolitinib’s anti-inflammatory properties to a Toll like receptor (TLR)3-mediated inflammatory response. In contrast to the data on human MDMs and LMs (generated in the present study) and a study of BMDMs from wild-type mice or IL-10 knock-out mice (FreitasMaria Cecilia et al., 2010; Pattison and MacKenzieElcombe, 2013), it has been reported that ruxolitinib and tofacitinib increase the LPS-induced production of TNFα, IL-6 and IL-12p40/70 in BMDMs from wild-type mice (Pattison and MacKenzie, 2012). The reasons for these discrepancies in mouse BMDMs have not been determined. Taken as a whole, however, the data suggest that ruxolitinib has a marked anti-inflammatory effect in mouse macrophages and other murine models of inflammation (Hazem et al., 2014).

Furthermore, the increase in LPS-induced cytokine production in wild-type mouse BMDMs was related to the inhibition of the IL-10 production by ruxolitinib; the anti-inflammatory IL-10 is critical in the negative feedback control of cytokine production in BMDMs (Pattison and MacKenzie, 2012). In the present study, however, IL-10 production by LMs was weakly inhibited by ruxolitinib—highlighting a major difference between mouse BMDMs and human LMs. It is also noteworthy that human monocytes, MDMs and macrophage cell lines are all surrogate cell models that do not adequately recapitulate the biology of human primary LMs (Martinez et al., 2013; Victoni et al., 2017). Indeed, we showed previously that β_2_-adrenoceptor agonists inhibited cytokine production by LPS-stimulated MDMs but not by LMs (Victoni et al., 2017); this finding promoted us to check ruxolitinib’s anti-inflammatory effects on LMs.

In human whole blood assays, ruxolitinib inhibited the JAK1 and JAK2 pathways with an IC_50_ ∼ 280 nM (Pattison and MacKenzieElcombe, 2013) in line with the submaximal inhibition of STAT3 phosphorylation at plasma concentration ∼ 1 µM (Chuglay et al., 2022). Therefore, the inhibitory effects of ruxolitinib on the LPS- and poly (I:C)-induced production of CXCL10 and on the poly (I:C)-induced production of TNF-α, IL-6, and CCL2 are likely related to JAK1/2 inhibition and clinically relevant.

JAK2 plays a pivotal role in LPS-induced signaling in macrophages (Okugawa et al., 2003). LPS immediately induces tyrosine phosphorylation of JAK2 *via* TLR4 and JAK2 regulates the phosphorylation of c-Jun NH2-terminal protein kinase (JNK) pathway, one of the mitogen-activated protein kinases’ pathways. Hence, JAK2 was involved in the LPS-induced production of IL-1β and IL-6 (Okugawa et al., 2003). It has been also reported that ruxolitinib was able to markedly suppress LPS-mediated induction of various human inflammatory cytokines (TNF-a, IL-6, CCL2, CXCL10), through antagonizing the autocrine interferon (IFN) β-related regulatory signalling pathway secondary triggered by LPS in human MDMs (Febvre-James et al., 2018).

### A comparison of ruxolitinib and budesonide

Budesonide was more potent than ruxolitinib in inhibiting LPS-induced cytokine production in LMs, with the exception of CXCL10. In fact, CXCL10 production was greater after budesonide treatment but was abrogated by low concentrations of ruxolitinib. It has been reported that corticosteroids do not suppress the CXCL10 production induced by LPS (alone or combined with IFN-γ) in human alveolar macrophages (Southworth et al., 2012; Armstrong et al., 2009a; Sargent and Singh 2009a). Ruxolitinib’s potent inhibition of CXCL10 production is an important finding because this chemokine recruits Th1 cells *via* the cognate receptor CXCR3 and thus contributes significantly to Th1-high asthma and failure to respond to corticosteroids (Gauthier et al., 2017). Furthermore, the CXCL10-CXCR3 signaling axis appears to contribute significantly to neutrophil-mediated lung injury and the development of both viral and non-viral acute respiratory distress syndromes (ARDSs) (Shan et al., 2017). In this respect, CXCL10 is considered to be a key immune factor in the cytokine storm observed in patients with coronavirus disease 2019 (COVID-19) and might be predictive of the clinical outcome (Zeiser et al., 2021).

Although ruxolitinib exerted anti-inflammatory effects on LPS- and poly (I:C)-stimulated LMs, it did not impair the cells’ phagocytic ability. Similarly, corticosteroids do not affect *in vitro* bacterial phagocytosis by human LMs (Martí-Lliteras et al., 2009; Higham et al., 2020). Given the phagolysosomal machinery’s roles in the innate and adaptive immune responses in general and the clearance of bacteria in particular, preservation of the LM’s phagocytic ability is essential.

### Ruxolitinib and the risk of infectious disease

Treatment with ruxolitinib has been variously linked to a greater risk of infection by intracellular pathogens [including reactivations of hepatitis B virus, herpes virus, and pulmonary *tuberculosis* (Tsukamoto et al., 2018; Khalid et al., 2021; Tzeng and Tsu Chyuan, 2021)], non-tuberculous mycobacteria, and opportunistic pathogens like *Pneumocystis jirovecii* and *Cryptococcus neoformans* (Wysham and Sullivan, 2013; Sayabovorn and Chongtrakool, 2021). Furthermore, *tuberculosis* reactivation *via* the “awakening” of dormant *Mycobacterium tuberculosis* has been linked to use of the JAK3/2 inhibitor tofacitinib (Maiga et al., 2015; Winthrop et al., 2016). In a recent report, the genes coding for IL-1β, TNF, IL-6, and JAK2 accounted for four of the seven most influential hub genes in the host response to *tuberculosis* disease (Alam et al., 2021). The *CXCL10* gene has also been identified as a hub gene that exhibits antituberculosis activity and is responsible for macrophage resistance against *M. tuberculosis* (Xu et al., 2021). Several monocyte-secreted cytokines (including IL-1β, IL-6, and TNF-α) and chemokines (including CXCL10) have been linked to the immunity induced by BCG vaccination and by mycobacterial growth inhibition (Joosten et al., 2018). In contrast, IL-1β in human macrophages was suppressed by virulent *M. tuberculosis* (Novikov et al., 2011), and it has been suggested that the suppression of CXCL10 production is a convergent immune evasion mechanism for intracellular pathogens (Antonia et al., 2019). Together with ruxolitinib’s inhibitory effect on the production of CXCL10 and (to a lesser extent) IL-6 and TNF-α by LMs, these data help to explain the greater risk of mycobacterial infections in patients treated with this JAK inhibitor.

### Study limitations and strengths

The present study’s main strength was the use of human primary LMs, rather than murine macrophages or human MDMs. The study’s main limitation was that the lung tissue samples were obtained from current/former smokers and patients with COPD. However, our primary LMs were sourced solely from macroscopically normal lung parenchyma located far from the tumor. Nevertheless, we cannot rule out a possible alteration in the LMs’ response to ruxolitinib in patients with a history of smoking or inflammatory disease. As discussed previously (Grassin-Delyle et al., 2013), the impact of smoking status and COPD on LPS-induced cytokine release by LMs varies markedly from one study to another. LPS-stimulated cytokine production by alveolar macrophages is reportedly higher in patients with COPD and smokers than in healthy non-smokers (Hodge et al., 2011). However, in a study of patients with COPD, there were no significant differences in cytokine production between current smokers and non-smokers (Hodge et al., 2011). In contrast, other researchers have found that a history of smoking was associated with lower levels of cytokine production by alveolar macrophages upon stimulation with LPS (Chen et al., 2007; Clark and Flanagan, 2014), and that current smoking status had no effect (i.e., the dose–response curves for the cytokines stimulated by LPS were similar in current smokers vs. former smokers) (Chen et al., 2007; Armstrong et al., 2011; Armstrong et al., 2009b, Sargent and Singh 2009b). Furthermore, the inhibitory effect of corticosteroids on the LPS-induced release of cytokines from LMs or alveolar macrophages isolated from bronchoalveolar lavages was similar 1) in non-smokers, current smokers and COPD patients, and 2) after a short (1 h) vs. a long (16 h) plate adherence step in the isolation procedure (Plumb et al., 2013; Higham et al., 2015; Higham et al., 2014). Hence, smoking status and COPD impair LPS-induced cytokine release to a variable extent but do not influence the inhibitory effect of corticosteroids on LMs. Therefore, the inhibitory effects of ruxolitinib observed in the present study are probably not restricted to LMs from ex-smokers or current smokers, and are likely to accurately reflect the *in vivo* responsiveness of human LMs. Furthermore, our alveolar macrophage preparations might have contained a small proportion of tissue-resident interstitial macrophages. However, exposure to bacterial and viral products [mimicked by LPS and poly (I:C) in the present study], inhaled corticosteroids or orally administered ruxolitinib is not restricted to the alveolar compartment, and the use of freshly isolated human LMs primarily from the alveolar spaces but perhaps also from interstitial tissue might provide a better picture of the clinical response.

The study’s second main limitation related to whether the ruxolitinib concentrations used here *in vitro* are clinically relevant. In fact, we carefully chose a concentration range that encompassed steady-state and peak plasma levels (∼10^–6^ M) of ruxolitinib observed in human patients on a normal treatment regimen, together with concentrations (∼10^–5^ M) previously reported to have a maximal effect on cytokine release by human monocytes or MDMs *in vitro* (Shilling et al., 2010; Lescoat et al., 2020).

Thirdly, the duration of exposure to ruxolitinib is limited to 24 h and we investigated the impact of drug solely on long life span fully differenciated pulmonary cells. This experimental protocol might fail to recapitulate the adequate context of a long-term exposure to the drug and/or regular recruitment of immature cells from the bone marrow related to intercurrent acute inflammatory flares.

### JAK inhibitors and inflammatory diseases of the airways

Inhibition of the JAK/STAT pathway might have clinical value in severe chronic inflammatory diseases of the airways in general (Georas et al., 2021; Matucci et al., 2021) and severe corticoresistant disease in particular (Barnes 2013, Barnes 2018; Subramanian et al., 2021). Furthermore, some literature data suggest that JAK inhibitors can improve the clinical outcome of hospitalized COVID-19 patients (Chen et al., 2021a; Chen et al., 2021b). However, these promising therapeutic targets should be considered with caution because ruxolitinib’s safety profile has not yet been fully defined—especially with respect to the risk of infectious adverse events (Winthrop 2017).

## Conclusion

Ruxolitinib exerted anti-inflammatory activity in LMs stimulated with LPS or poly (I:C), without impairing the cells’ phagocytic activity. The drug’s observed anti-inflammatory action (particularly its ability to inhibit production of the corticosteroid-resistant chemokine CXCL10 by LMs) suggests that add-on treatment with a JAK inhibitor might be a valuable anti-inflammatory strategy in chronic obstructive pulmonary disease, Th1-high asthma, and both viral and *via* non-viral ARDSs (including COVID-19).

## Data Availability

The raw data supporting the conclusion of this article will be made available by the authors, without undue reservation.

## Abbreviations

ARDS, acute respiratory distress syndromes; BMDM, bone-marrow-derived macrophage; CCL, C-C motif ligand; CXCL, C-X-C motif ligand; COPD, chronic obstructive pulmonary disease; FEV1, Forced Expiratory Volume in 1 s; FVC, Forced Vital Capacity; IL, interleukin; JAK, Janus kinase; LM, lung macrophage; LPS, lipopolysaccharide; MDM, monocyte-derived macrophage; RPMI, Roswell Park Memorial Institute; STAT, signaling transducer and activator of transcription proteins; TLR, Toll Like Receptor; TNF-α, tumor necrosis factor alpha.
