# Supplementary material for: Ruxolitinib inhibits cytokine production by human lung macrophages without impairing phagocytic ability
Source: Front Pharmacol. 2022 Aug 19;13:896167. doi: 10.3389/fphar.2022.896167 (PMC9437255; doi:10.3389/fphar.2022.896167)
Supplement: Supplementary file 1 [file DataSheet1.pdf]

Supplemental Data

Ruxolitinib inhibits cytokine production by human lung macrophages without impairing phagocytic ability

Assessment of cytotoxicity of ruxolitinib

The cytotoxicity of ruxolitinib was determined in two different ways

- measuring the lactate dehydrogenase activity in the LM supernatants, using the CytoTox96® Non-Radioactive Cytotoxicity Assay (Promega, Madison, WI) after 24 h of exposure to ruxolitinib at the highest dose (10^{-5} M).
  The percentage of LDH release in the supernatants by LMs incubated with ruxolitinib compared to unstimulated LMs was not significantly different, neither was the one from LMs stimulated by LPS or Poly (I:C) alone (Table S1)

  Table S1. Ruxolitinib effect on LDH release by LMs

	% of LDH release *versus* control	
	mean	s.e.m
Ruxolitinib 10-5 M	-0.73	0.61
LPS 10 ng/ml	-0.41	0.12
Ploy (I:C) 10 µg/ml	0.1	0.11

  LMs were incubated in presence of ruxolitinib (10-5 M), LPS 10 ng/ml) or Poly (I:C) (10 µg/ml) or left unstimulated during 24h before recovering the supernatants. The level of LDH enclosed in the supernatants of treated LMs was measured using the CytoTox96® Non-Radioactive Cytotoxicity Assay (Promega, Madison, WI) and reported to controls. The mean ± SEM results of 3 independent experiments are expressed in % of LDH release versus control (resting LMs)

- Staining live cells with a Live/dead flow cytometry staining.

LMs were cultured with ruxolitinib during 24 hours before being gently detached, stained with a LIVE/DEAD Fixable Aqua Dead Cell Stain Kit (ThermoFisher Scientific, Waltham, MA) and analyzed by flow cytometry. The percentage of live cells was consistently over 90%, mean of 93.1 ±2.5 % (n=5).

<u>Figure S1. Live/dead staining on LMs</u>

Gating strategy and mean results obtained from 5 distinct experiments

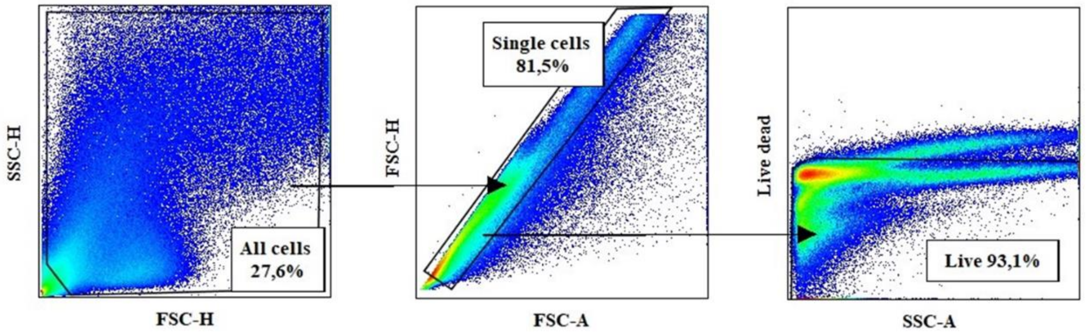

Table S2. Ruxolitinib's effect on the LPS-induced cytokine production by LMs

The mean ± SEM results of five to 12 independent experiments are expressed in pg.10^{-6} LMs. Asterisks indicate significant differences relative to the LPS condition (*: <0.05; **: <0.01; ***: <0.001). Ruxolitinib's mean inhibitory effect on the LPS-induced cytokine production is expressed as a percentage of the response to LPS alone and is given in square brackets.

	LPS *10 ng.ml^{-1}*	*Ruxolitinib* *5x10^{-7} M*	*Ruxolitinib* *10^{-6} M*	*Ruxolitinib* *5x10^{-6}M*	*Ruxolitinib* *10^{-5} M*
**IL-1β** n=5	431.2±133.2	372.9±176.3	288.2±105.2	237.1±79.1	265.1±62.5
**TNF-α** n= 12	57273.8±7321.6	59762.2±8525	60542.6±7848.6	49464.9±6975.9	38172.5±6185.3 [-33%] ***
**IL-6** n=10	74390.7±16518.9	70277.5±12306.9	62193.7±11261.4	61011.8±14238.5	57274.3±16777.2 [-33%] ***
**CCL2** n=11	33431.6±8147.1	16282.5±5201.2 [-58%] *	14695.5±5281.2 [-62%] *	9543.5±3660.6 [-78%] ***	6062±2176.2 [-86%] ***
**CXCL10** n=9	3623.6±1174.1	99.9±37.2 [-92%] **	70.6±19 [-96%] ***	84.7±26.1 [-89%] *	87.6±30.7 [-90%] *
**IL-10** n=8	2531.7±286	1785.5±262.4 [-28%] *	1412.2±160.1 [-40%] *	1331±183.7 [-44%] *	1041.2±172.8 [-57%] *

Table S3. Ruxolitinib's effect on poly(I:C)-induced cytokine production by LMs

The mean ± SEM results of five to eight independent experiments are expressed in pg.10^{-6} LMs. Asterisks indicate significant differences relative to poly(I:C) (*: <0.05; **: <0.01; ***: <0.001). Ruxolitinib's inhibitory effect on poly(I:C)-induced cytokine production is expressed as a percentage of the response to poly(I:C) alone and is given in square brackets.

	poly(I:C) *1 µg.mL^{-1}*	*Ruxolitinib* *5x10^{-7} M*	*Ruxolitinib* *10^{-6} M*	*Ruxolitinib* *5x10^{-6}M*	*Ruxolitinib* *10^{-5} M*
**IL-1β** n=3	1179.2±227.5	916.6±57.7	826.1±62	914.9±125.4	398.6±39.4 [-64%] **
**TNF-α** n= 8	9336.5±2651.1	5175.1±2481.3	3859.5±2173.9	4698±3860.6 [-67%] *	2070.9±1312.6 [-80%] ***
**IL-6** n=8	13859.7±2811.7	8468.4±2903.3	7057.7±2458.1 [-51%] *	6407.6±2672.3 [-57%] *	6932.8±3093.2 [-58%] **
**CCL2** n=8	14841.8±4409.2	9939.7±3802.6	7389.3±3260.9	7344.7±4319.8 [-55%] *	2761.5±897.2 [-77%] ***
**CXCL10** n=7	8266±1568.9	3650.1±1815.3	495±400.2 [-95%] *	68.6±23.9 [-98%] ***	77.4±25.4 [-98%] ***
**IL-10** n=5	671.9±124.5	505.9±132.9	477.5±31	464.9±137.7	315.5±100.1 [-59%] **

Table S4. Budesonide' effect on LPS-induced cytokine production by LMs

The mean ± SEM results of 5 to 10 independent experiments are expressed in pg.10^{-6} LMs. Asterisks indicate significant differences, relative to LPS alone (*: <0.05; **: <0.01; ***: <0.001). Budesonide's inhibitory effect on LPS-induced cytokine production is expressed as a percentage of the response to LPS alone and is given in square brackets.

	LPS 10 ng.ml^{-1}	*Budesonide 10^{-11} M*	*Budesonide 10^{-10} M*	*Budesonide 10^{-9} M*	*Budesonide 10^{-8} M*
**IL-1β** n=10	262.3±93.8	286.2±104.8	241.6±96.6	179.1±72.4	123.4±61.1 [-44%] **
**TNF-α** n= 5	34096.2±6721.2	26223.6±5585	20432.8±4105 [-40%] *	8952.3±1956.6 [-73%] ***	2224.6±478.3 [-94%] ***
**IL-6** n=6	42875.3±8028.3	43090±7067.4	34517±8890.3	16838.5±3359.9 [-59%] *	3784.9±1052.6 [-88%] **
**CCL2** n=5	35370±9247.6	32738.7±11538.3	24649.6±6852.5	13256.4±4647.1 [-65%] ***	7106.1±3980.7 [-84%] ***
**CXCL10** n=5	4128.4±1780.2	3232.4±1342.2	3442.2±1394.8	6118.4±2608.6	5319.5±1911.8
**IL-10** n=8	634.2±164.2	687.7±119.1	483.3±101.4	310.1±56.9	83.9±18.2 [-81%] ***
